# Supplementary material for: Analysis of PPARγ Signaling Activity in Psoriasis
Source: Int J Mol Sci. 2021 Aug 10;22(16):8603. doi: 10.3390/ijms22168603 (PMC8395241; doi:10.3390/ijms22168603)
Supplement: Supplementary file 1 [file ijms-22-08603-s001.zip › Supplemental materials_Analysis of PPARg signaling activity in psoriasis/Pathway models/Models images and html files/Interleukin-17 and interleukin-22 signaling in psoriasis/9338.html]

MAP2K3


# Protein MAP2K3

|  |  |
| --- | --- |
| URN | urn:agi-llid:5606 |
| Total Entities | 0 |
| Connectivity | 809 |
| Name | MAP2K3 |
| Description | mitogen-activated protein kinase kinase 3 |
| Notes | The protein encoded by this gene is a dual specificity protein kinase that belongs to the MAP kinase kinase family. This kinase is activated by mitogenic and environmental stress, and participates in the MAP kinase-mediated signaling cascade. It phosphorylates and thus activates MAPK14/p38-MAPK. This kinase can be activated by insulin, and is necessary for the expression of glucose transporter. Expression of RAS oncogene is found to result in the accumulation of the active form of this kinase, which thus leads to the constitutive activation of MAPK14, and confers oncogenic transformation of primary cells. The inhibition of this kinase is involved in the pathogenesis of Yersina pseudotuberculosis. Multiple alternatively spliced transcript variants that encode distinct isoforms have been reported for this gene. [provided by RefSeq, Jul 2008] |
| Primary Cell Localization | Cytoplasm |
| Class | Protein kinase |
| ObjectType | Protein |

---

|  |  |
| --- | --- |
| Pathway | Osteoarthritis |
|  | Skin Aging |
|  | Dopamine Receptors Signaling |
|  | Serotonin Receptors Signaling |
|  | Toll-like Receptors Act through MYD88-TIRAP Signaling |
|  | TLR4 Signaling in Leukocytes |
|  | T-Cell Dependent B-Cell Activation |
|  | Plasmin Effects in Inflammation |
|  | Bradykinin Effects in Inflammation |
|  | Toll-like Receptors in Sterile Inflammation |
|  | Leukotriene Effect on Vascular Endothelial Cell Response |
|  | Mast-Cell Activation without Degranulation through TNFSF8 Signaling |
|  | Mast-Cell Activation without Degranulation |
|  | Mast-Cell Activation without Degranulation through IL33/IL1RL1 Signaling |
|  | Mast-Cell Activation without Degranulation through IL1R1 and TLR Signaling |
|  | AXL Receptor Inhibits Macrophages and Dendritic Cells Function |
|  | Neutrophil Recruitment and Priming |
|  | Vascular Smooth Muscle Cell/Pericyte Migration and Proliferation |
|  | Vascular Endothelial Cell Activation by Cytokines |
|  | Vascular Endothelial Cell Activation by Growth Factors |
|  | GFs/TNF -> Ion Channels |
|  | Nociception Expression Targets Signaling |
|  | EDNRA/B -> Vascular Motility |
|  | ADRA1A -> IL6 Production |
|  | ADRA1 -> Prostaglandin Generation |
|  | BDKRB1/2 -> Prostaglandin Generation |
|  | CNR1/2 -> IL1B/2/4/6/10 Production |
|  | DRD2 Expression Targets |
|  | CHRM1 -> IL2 Production |
|  | HTR1 -> IL6 Production |
|  | HTR5 -> TNF Production |
|  | HTR1 -> Membrane Transport |
|  | IL1B Expression Targets -> Nociception |
|  | Circadian Clock Genes in Suprachiasmatic Nuclei Neurons |
|  | Cardiomyocyte Dysfunction |
|  | Dendritic Cells Function in Atherosclerosis |
|  | Proteins Involved in Dilated Cardiomyopathy |
|  | Cardiomyocyte Hypertrophy |
|  | Hypertrophic Cardiomyopathy |
|  | Myocardial Remodeling |
|  | Myocardial Infarction |
|  | Inflammatory Reaction in Acne Vulgaris |
|  | Keratinocyte Proliferation in Acne Vulgaris |
|  | Mast-Cells Activation in Atopic Dermatitis |
|  | IL17 Signaling in Psoriasis |
|  | Dendritic Cells Function in Psoriasis |
|  | B-Cell Activation in Systemic Lupus Erythematosus |
|  | Humoral Immunity in Vitiligo |
|  | Podocyte Dysfunction in Diabetic Nephropathy |
|  | Mesangial Cell Dysfunction in Diabetic Nephropathy |
|  | Adiponectin in Insulin Resistance Prevention |
|  | Oxidation of Fatty Acid in non-Alcoholic Fatty Liver Disease |
|  | beta-Cell Destruction through Cytokines in Diabetes Mellitus |
|  | Dendritic Cell Function in Ulcerative Colitis |
|  | Epithelial Cell in the Innate Immune Response in Ulcerative Colitis |
|  | Osteoclast Activation in Multiple Myeloma |
|  | Hepatocellular Carcinoma |
|  | Growth Factor Signaling in Hepatocellular Carcinoma |
|  | Pancreatic Ductal Carcinoma |
|  | Growth Factor Signaling in Pancreatic Neoplasms |
|  | IL33 Signaling Related Eosinophilia in Asthma |
|  | TSLP Signaling in Bronchial Epithelial Cell in Asthma |
|  | Mucin Production in Goblet Airway Epithelial Cells |
|  | CFTR Facilitates Oxidative Stress in Airway Epithelium in CF |
|  | Airway Epithelial Cell Dysfunction |
|  | CFTR Expression in Epithelial Cells (Class I Mutations) |
|  | Osteoblast Function Decline in Gout |
|  | Monocytes Function in Gout |
|  | TNF and IL1B Induce Metalloproteinase Synthesis in Osteoarthritis |
|  | Apoptosis of Chondrocyte |
|  | IL1B Induced Arthralgia |
|  | TGFB2 Signaling Impairement in Osteoarthritis |
|  | Dendritic Cell Dysfunction in Psoriatic Arthritis |
|  | Synovial Fibroblast Activation in Psoriatic Arthritis |
|  | Synovial Fibroblast Activation by Citokines in Rheumatoid Arthritis |
|  | Apoptosis Block in Synovial Fibroblast |
|  | TLR2 Induced Synovial Fibroblast Activation in Rheumatoid Arthritis |
|  | Autocrine Cytokine/Chemokine Loops in Systemic Scleroderma |
|  | B-Cells Function in Systemic Scleroderma |
|  | Skin Fibrosis |
|  | CCR5 Signaling in Macrophages |
|  | Apoptosis Block in Infected Cells |
|  | Diabetes Induced Periodontitis |
|  | IL1 Receptor Antagonist (IL1RN) Deficiency (DIRA) |
|  | IL36 Receptor Antagonist (IL1F5) Deficiency (DITRA) |
|  | Majeed Syndrome |
|  | Bone Loss in Osteoporosis |
|  | Hyperparathyroidism, Secondary Effect |
|  | Glioma Invasion Signaling |
|  | Growth Factor Signaling in Neuroblastoma |
|  | Neuroblastoma |
|  | Microglia and Motor Neuron Interaction Dysregulation |
|  | Crystallin alpha B (CRYAB) Suppresses Multiple Sclerosis |
|  | ESR1/ERBB Positive Luminal Breast Cancer |
|  | Breast Cancer |
|  | ESR1 Signaling in Breast Cancer |
|  | Endometrial Cancer |
|  | Endometrioid Endometrial Cancer |
|  | Clear Cell Endometrial Cancer and Papillary Serous Endometrial Cancer |
|  | Angiogenesis in Endometriosis |
|  | Endometrioid Ovarian Carcinoma |
|  | Clear Cell Ovarian Carcinoma |
|  | High-Grade Serous Ovarian Carcinoma |
|  | Low-Grade Serous Ovarian Carcinoma |
|  | Ovarian Cancer |
|  | Mucinous Ovarian Carcinoma |
|  | Subepithelial Fibroblasts in Middle Ear Cholesteatoma |
|  | Cholesteatoma |
|  | HPV E5 Regulates EDNRA and EGFR Signaling Pathway in Keratinocytes |
|  | Androgen Receptor/Akt Signaling |
|  | Steroids Induced Cataract |
|  | VEGFA in Neovascular Glaucoma |
|  | Neurotrophic Factor Deprivation in Retinal Ganglion Cell Death |
|  | Cytokines Trigger Otitis Media |
|  | MUC5AC Expression by NTHi and S. Pneumoniae |
|  | Immune System Role in Otitis Media |
|  | Alveolar Macrophages Dysfunction in COPD |
|  | T-Cell Cytotoxic Activation |
|  | Alveolar Epithelial Cell Dysfunction in COPD |
|  | Mucin Hyperproduction in Goblet and Mucous Cells |
|  | Plasmin Role in Glomerulonephritis |
|  | Endothelial Cell Dysfunction in Glomerulonephritis |
|  | Podocyte Dysfunction in Glomerulonephritis |
|  | Mesangial Cell Dysfunction in Glomerulonephritis |
|  | Interstitial Fibroblasts in Pyelonephritis |
|  | Apoptosis of Renal Tubule Epithelial Cell in Pyelonephritis |
|  | ANGPT1 -> CREB/CREBBP Expression Targets |
|  | ANGPT2 -> AP-1/CREBBP/MYC Expression Targets |
|  | AREG -> NFATC Expression Target |
|  | AREG -> CREB/CREBBP Expression Targets |
|  | AREG -> AP-1 Expression Targets |
|  | Acetylcholine Expression Targets |
|  | Dopamine/Gi Expression Targets |
|  | BTC -> NFATC Expression Targets |
|  | BTC -> AP-1/ATF/CREB Expression Targets |
|  | Frizzled Receptors -> ARRB1/ARRB2 non-Canonical Signaling |
|  | Sialophorin -> CTNNB/MYC/TP53 Signaling |
|  | CD72 -> NF-kB Expression Targets |
|  | CD8 -> NF-kB Expression Targets |
|  | CD80 -> NFATC Expression Targets |
|  | CD86 -> NFATC Expression Targets |
|  | Clozapine Induced Granulocytopenia |
|  | EGF -> AP-1/ATF Expression Targets |
|  | EGF -> CREB/CREBBP/ELK/SRF/MYC Expression Targets |
|  | EGF -> MEF/MYOD/NFATC Expression Targets |
|  | EGF -> TP53 Expression Targets |
|  | EGFR/ERBB3 -> MEF/MYOD/NFATC/MYOG Signaling |
|  | EGFR/ERBB2 -> TP53 Signaling |
|  | EGFR -> AP-1/ATF2 Signaling |
|  | EGFR -> AP-1/CREB/ELK/SRF/MYC Signaling |
|  | EREG -> CREB Expression Target |
|  | EREG -> AP-1/ATF Expression Targets |
|  | Erythropoietin -> AP-1/MYC/CREB Expression Targets |
|  | F2 -> AP-1/CREB/ELK/SRF/SP1 Expression Targets |
|  | PLG -> AP-1/CREB/ELK/SRF/SP1 Expression Targets |
|  | FGF9 -> AP-1/CREB/MYC Expression Targets |
|  | FGF10 -> AP-1/CREB/CREBBP/MYC Expression Targets |
|  | FGF1 -> AP-1/CREB/ELK/SRF/MYC Expression Targets |
|  | FGF8 -> AP-1/CREB/MYC Expression Targets |
|  | FGF18 -> AP-1/CREB Expression Targets |
|  | FGF4 -> AP-1/MYC Expression Targets |
|  | FGF7 -> AP-1/CREB/CREBBP/MYC Expression Targets |
|  | FGF2 -> AP-1/CREB/CREBBP/ELK/SRF/MYC Expression Targets |
|  | FGFR -> AP-1/CREB/CREBBP/ELK/SRF/MYC Signaling |
|  | ADCYAP1 Expression Targets |
|  | Glutamate/Gq Expression Targets |
|  | VIP Expression Targets |
|  | HBEGF -> AP-1/ATF Expression Targets |
|  | HBEGF -> TP53 Expression Targets |
|  | HBEGF -> MEF/MYOD Expression Target |
|  | HBEGF -> CREB/MYC Expression Targets |
|  | HGF -> AP-1/CREB/ELK/SRF/MYC Expression Targets |
|  | HGFR -> AP-1/CREB/MYC Signaling |
|  | TGFB Signaling activation by Blocking of Tumor Suppressors |
|  | TGFB Family in Epithelial to Mesenchymal Transition in Cancer |
|  | IGF1 -> MEF/MYOD/MYOG Expression Targets |
|  | IGF2/MEF/MYOD Expression Targets |
|  | Insulin -> MEF/MYOD Expression Targets |
|  | ICAM1 -> AP-1/CREB/ELK/SRF Signaling |
|  | IGF1R -> MEF/MYOD/MYOG Signaling |
|  | FibronectinR -> NF-kB Signaling |
|  | FibronectinR -> AP-1/ELK/SRF/SREBF Signaling |
|  | Fibronectin Expression Targets |
|  | CTGF -> AP-1/CREB/MYC Expression Targets |
|  | ICAM1 Expression Targets |
|  | IL1R -> NF-kB Signaling |
|  | IL16-> ATF/CREB/CREBBP Expression Target |
|  | PAF/Gq -> AP-1/ATF1/CREB/ERK/SRF Expression Targets |
|  | Dronabinol/Anandamide Expression Targets |
|  | PAF/Gq -> NF-kB Expression Targets |
|  | S1P Expression Targets |
|  | NRG1 -> MEF/MYOD Expression Targets |
|  | NRG1 -> CREB/CREBBP/ELK/SRF/MYC Expression Targets |
|  | NRG1 -> TP53 Expression Target |
|  | NRG1 -> AP-1/ATF Expression Targets |
|  | NGF -> SMAD3/NF-kB Expression Targets |
|  | NGF -> AP-1/TP53/MYC Expression Targets |
|  | BDNF Expression Targets |
|  | NTF3 Expression Targets |
|  | NTF4 Expression Targets |
|  | NGF -> CREB/CEBPB/MEF2A Expression Targets |
|  | NOTCH -> TCF3 Signaling |
|  | NOTCH Expression Targets |
|  | PDGFB -> AP-1/CREB/MYC Expression Target |
|  | PDGFC -> CREB Expression Target |
|  | PDGFD -> AP-1 Expression Targets |
|  | PDGF -> AP-1/CREB/CREBBP/MYC Expression Targets |
|  | PDGFR -> AP-1/MYC Signaling |
|  | AVP/Gs -> CREB/ELK/SRF/AP-1/EGR Expression Targets |
|  | EDN1 Expression Targets |
|  | AVP/Gs -> MEF/MYOD/NFATC/MYOG Expression Targets |
|  | AVP/Gq -> MEF/MYOD/NFATC/MYOG Expression Targets |
|  | EDN3 Expression Targets |
|  | AVP/Gq -> CREB/ELK/SRF/AP-1/EGR Expression Targets |
|  | PDCD1 -> ATF/CREB/CREBBP Expression Targets |
|  | AngiopoietinR -> AP-1 Signaling |
|  | NTRK -> AP-1/CREB/ELK/SRF/MYC/SMAD3/TP53 Signaling |
|  | GAS6 -> AP-1/CREB Expression Targets |
|  | FIGF -> AP-1 Expression Target |
|  | KITLG -> AP-1/CREB/CREBBP/MYC Expression Targets |
|  | CSF1 -> AP-1/CREB/CREBBP/MYC Expression Targets |
|  | PGF -> AP-1/CREB/CREBBP/MYC Expression Targets |
|  | FLT3LG -> AP-1/CREB/CREBBP Expression Targets |
|  | TGFBR -> AP-1 Signaling |
|  | TGFBR -> CREB/ELK-SRF Signaling |
|  | TGFBR -> ATF/GADD/MAX/TP53 Signaling |
|  | TGFBR -> MEF/MYOD/MYOG Signaling |
|  | TNF-alpha/TNFRSF1A Signaling |
|  | TGF-beta Signaling |
|  | BMPR2 Signaling |
|  | P38 MAPK/MAPK14 Signaling |
|  | TNF-alpha/TNFRSF1B Signaling |
|  | VEGF Signaling |
|  | TCR -> CREB/CREBBP/ATF Expression Targets |
|  | T-Cell Receptor -> ATF/CREB Signaling |
|  | TGFA -> TP53 Expression Targets |
|  | TGFA -> MEF/MYOD/NFATC Expression Targets |
|  | TGFA/AP-1/ATF Expression Targets |
|  | TGFA -> CREB/CREBBP/ELK-SRF/MYC Expression Targets |
|  | TGFB3-TGFBR2 Expression Targets |
|  | TGFB2-TGFBR2 Expression Targets |
|  | TGFB1-TGFBR2 Expression Targets |
|  | FASLG Expression Targets |
|  | TNF -> NF-kB Expression Targets |
|  | TNFSF10 Expression Targets |
|  | LTA Expression Targets |
|  | TNFSF14 Expression Targets |
|  | TNF -> AP-1 Expression Targets |
|  | CD72 -> AP-1 Expression Targets |
|  | CD40LG -> NF-kB/ELK/SRF -> CREB/NFATC Expression Targets |
|  | TNFSF13 Expression Targets |
|  | TNFSF13B Expression Targets |
|  | TNF -> ELK-SRF Expression Targets |
|  | TNF -> CREB Expression Targets |
|  | TNF -> TP53/ATF Expression Targets |
|  | EDA Expression Targets |
|  | EctodysplasinR -> NF-kB Signaling |
|  | TNFR -> CREB/ELK-SRF Signaling |
|  | TNFR -> AP-1/ATF/TP53 Signaling |
|  | NGFR -> AP-1/CEBPB/CREB/ELK/SRF/TP53 Signaling |
|  | TNFRSF5/6 -> RB1/E2F Signaling |
|  | NGFR -> NF-kB Signaling |
|  | TNFR -> NF-kB Signaling |
|  | NGFR -> MEF Signaling |
|  | TNFRSF6 -> DDIT3 Signaling |
|  | TNFRSF1A -> AP-1/ATF/TP53 Signaling |
|  | TNFRSF1A -> CREB/ELK-SRF Signaling |
|  | Thrombopoietin -> AP-1/CREB/CREBBP/MYC Expression Targets |
|  | TLR4 -> AP-1 Expression Targets |
|  | TLR5 Expression Targets |
|  | TLR7 Expression Targets |
|  | TLR4 -> AP-1/EGR1/HIF1A Expression Targets |
|  | TLR1 -> 2/6 Expression Targets |
|  | IL1B Expression Targets |
|  | IL1A Expression Targets |
|  | TLR9 Expression Targets |
|  | TLR -> AP-1 Signaling |
|  | ER Stress (Unfolded Protein Response) |
|  | ROS in Angiotensin Mediated Cardiovascular Remodeling and Hypertrophy |
|  | VEGFA -> ATF/CREB/ELK-SRF Expression Targets |
|  | VEGFA -> NFATC Expression Targets |
|  | VEGFC -> ATF Expression Target |
|  | VEGFA -> AP-1/CREBBP/MYC Expression Targets |
|  | VEGFR -> NFATC Signaling |
|  | VEGFR -> AP-1/CREB/MYC Signaling |
|  | VEGFR -> ATF/CREB/ELK-SRF Signaling |
|  | pathway 4 |
|  | Model of PPARG signaling in psoriasis |
|  | Model of PPARG related pathways in psoriasis (short version) |
|  | dsRNA induces innate immunity and INFs in alveolar cells |
|  | 1\_Inflammation in keratinocytes in the pathology of acne |
|  | 2\_Interleukin-17 and interleukin-22 signaling in psoriasis |
|  | 1\_TNF and IL1B provoke ECM degradation in osteoarthritis |
|  | 1\_Osteoclast functional impairment in osteopetrosis |
|  | 2\_Chondrocyte death in osteoarthritis |
|  | 3\_The role of interstitial fibroblasts in pyelonephritis |
|  | 3\_Mesangial cell dysfunction in glomerulonephritis |
|  | 2\_Podocyte dysfunction in glomerulonephritis |
|  | 3\_Role of neurotrophic factor deprivation in retinal ganglion cell death |
|  | 2\_Pathogens stimulate mucins expression in the middle ear |
|  | 3\_Arterial wall inflammation and calcification in atherosclerosis |
|  | 2\_Cardiomyocyte hypertrophy |
|  | 3\_Cardiomyocyte dysfunction unrelated to cell junctions in ARVD |
|  | 1\_Cigarette smoke causes dysfunction and production of inflammatory mediators in alveolar epithelial cells |
|  | 4\_Inflammatory mediators inhibit GATA signaling in erythropoiesis |
|  | 4\_2\_Insulin resistance: inflammation-related insulin resistance |
|  | 4\_3\_Insulin resistance: FFA-related insulin resistance |
|  | 2\_Neuron and Schwann cell death |
|  | Model of PPARG signaling in psoriais (tested) |
|  | mapk1 protein modificators |
|  | Interleukin-17 and interleukin-22 signaling in psoriasis |

---

|  |  |
| --- | --- |
| Source | curated |

---

|  |  |
| --- | --- |
| MedScan ID | 5606 |

---

|  |  |
| --- | --- |
| LocusLink ID | 5606 |
|  | 26397 |
|  | 303200 |
|  | 100911550 |
|  | 19103 |
|  | 104607 |
|  | 365239 |
|  | 65239 |

---

|  |  |
| --- | --- |
| Alias | MKK3 |
|  | MAP kinase kinase 3 |
|  | SAPKK2 |
|  | dual specificity mitogen activated protein kinase kinase 3 |
|  | MAPK kinase 3 |
|  | OTTMUSP00000006687 |
|  | Dual specificity mitogen-activated protein kinase kinase III |
|  | LOC92079 |
|  | MKK3b |
|  | LOC82070 |
|  | mMKK3b |
|  | MAP kinase kinase III |
|  | AW212142 |
|  | OTTHUMP00000166044 |
|  | MAPKK3 |
|  | MEK3 |
|  | stress-activated protein kinase kinase 2 |
|  | mitogen activated protein kinase kinase 3 |
|  | RP23-467E19.1 |
|  | MAPK/ERK kinase III |
|  | mitogen-activated protein kinase kinase III |
|  | Map2k3 |
|  | MAPK kinase III |
|  | SAPK kinase 2 |
|  | PRKMK3 |
|  | LOC303200 |
|  | LOC100911550 |
|  | MAPK/ERK kinase 3 |
|  | SAPKK-2 |
|  | MAPKK 3 |
|  | MEK 3 |
|  | dual specificity mitogen-activated protein kinase kinase 3 |
|  | dual specificity mitogen-activated protein kinase kinase 3-like |
|  | SKK2 |
|  | protein kinase, mitogen-activated, kinase 3 |
|  | mitogen-activated protein kinase kinase 3 |
|  | MAPKK3s |
|  | PRKMK3s |
|  | protein kinase, mitogen-activated, kinase III |
|  | kinase, mitogen-activated, kinase |
|  | Map2k3\_predicted |
|  | Fam32a\_predicted |

---

|  |  |
| --- | --- |
| EC Number | 2.7.12.2 |
|  | 2.7.1.- |

---

|  |  |
| --- | --- |
| GO ID | 0005524 |
|  | 0004708 |
|  | 0019901 |
|  | 0004674 |
|  | 0004713 |
|  | 0000187 |
|  | 0032147 |
|  | 0060048 |
|  | 0035924 |
|  | 0006954 |
|  | 0038066 |
|  | 0018108 |
|  | 0043536 |
|  | 0045860 |
|  | 0045893 |
|  | 0042035 |
|  | 0007165 |
|  | 0023014 |
|  | 0031098 |
|  | 0005737 |
|  | 0005829 |
|  | 0016020 |
|  | 0005654 |
|  | 0016301 |
|  | 0000166 |
|  | 0004672 |
|  | 0016740 |
|  | 0000165 |
|  | 0016310 |
|  | 0071222 |
|  | 0072709 |
|  | 0007507 |
|  | 0051770 |
|  | 0001934 |
|  | 0002931 |
|  | 0005622 |
|  | 0045087 |
|  | 0034146 |
|  | 0051403 |
|  | 0035556 |
|  | 0034142 |
|  | 0002756 |
|  | 0002755 |
|  | 0004702 |
|  | 0005515 |
|  | 0034134 |
|  | 0034138 |
|  | 0035666 |
|  | 0008063 |
|  | 0034130 |
|  | 0009405 |
|  | 0034166 |
|  | 0035897 |
|  | 0038123 |
|  | 0016772 |
|  | 0038124 |
|  | 0034162 |
|  | 0007346 |
|  | 0006468 |
|  | 0002224 |
|  | 0003674 |
|  | 0005575 |

---

|  |  |
| --- | --- |
| KEGG ID | hsa:5606 |
|  | mmu:26397 |
|  | rno:303200 |

---

|  |  |
| --- | --- |
| MedLine Reference | 10097111 |
|  | 10202148 |
|  | 12466851 |
|  | 8900184 |
|  | 11980910 |
|  | 7839144 |
|  | 12477932 |
|  | 14709549 |
|  | 10349636 |
|  | 10922068 |
|  | 11042159 |
|  | 11076861 |
|  | 11217851 |
|  | 11447117 |
|  | 12151339 |
|  | 12374793 |
|  | 12893778 |
|  | 14633627 |
|  | 14657222 |
|  | 15143069 |
|  | 15644321 |
|  | 15767678 |
|  | 15778378 |
|  | 15782199 |
|  | 16141072 |
|  | 16141073 |
|  | 16199873 |
|  | 16357362 |
|  | 16567640 |
|  | 17030606 |
|  | 8889548 |
|  | 10497253 |
|  | 10856288 |
|  | 10997882 |
|  | 11062067 |
|  | 11279118 |
|  | 11279172 |
|  | 11971971 |
|  | 12024021 |
|  | 12054652 |
|  | 12377770 |
|  | 12589052 |
|  | 13679851 |
|  | 14702039 |
|  | 15375157 |
|  | 15516490 |
|  | 15677464 |
|  | 15778394 |
|  | 15797859 |
|  | 16344560 |
|  | 16709574 |
|  | 7750576 |
|  | 8226933 |
|  | 8622669 |
|  | 8626699 |
|  | 9038352 |
|  | 9373149 |
|  | 9465908 |
|  | 9768359 |
|  | 9786855 |
|  | 9841871 |
|  | 10995439 |
|  | 16109975 |
|  | 11181995 |
|  | 11414763 |
|  | 15489334 |
|  | 16728640 |
|  | 17406030 |
|  | 17438131 |
|  | 8125298 |
|  | 11593045 |
|  | 14634666 |
|  | 16237070 |
|  | 16365149 |
|  | 17167049 |
|  | 17214902 |
|  | 17588539 |
|  | 17723219 |

---

|  |  |
| --- | --- |
| Organism | Homo sapiens {Organism urn:agi-taxid:9606} |
|  | Mus musculus {Organism urn:agi-taxid:10090} |
|  | Rattus norvegicus {Organism urn:agi-taxid:10116} |
|  | Homo sapiens |
|  | Mus musculus |
|  | Rattus norvegicus |
|  | Danio rerio |

---

|  |  |
| --- | --- |
| Mouse chromosome position | 11 |
|  | 11 B2 |

---

|  |  |
| --- | --- |
| OMIM ID | 602315 |
|  | OMIM:602315 |

---

|  |  |
| --- | --- |
| Rat chromosome position | 10q22 |
|  | 10 |

---

|  |  |
| --- | --- |
| Hugo ID | 6843 |
|  | HGNC:6843 |

---

|  |  |
| --- | --- |
| Human chromosome position | 17p11.2 |
|  | 17q11.2 |

---

|  |  |
| --- | --- |
| Swiss-Prot Accession | P46734 |
|  | Q6FI23 |
|  | P46734.2 |
|  | O09110 |
|  | Q5SWN9 |
|  | O09110.2 |
|  | B1H230 |
|  | Q498S1 |
|  | B3KSK7 |
|  | Q99441 |
|  | Q9UE71 |
|  | Q9UE72 |
|  | P97293 |
|  | Q91VX1 |
|  | Q53EZ9 |
|  | Q6FHG1 |
|  | Q3TJG7 |
|  | Q3TLX7 |
|  | Q3TX82 |
|  | Q3UDI2 |
|  | MP2K3\_HUMAN |
|  | MP2K3\_MOUSE |
|  | Q6FI23\_HUMAN |
|  | B1H230\_RAT |
|  | Q5SWN9\_MOUSE |

---

|  |  |
| --- | --- |
| GenBank ID | NC\_000017 |
|  | XM\_017024859 |
|  | XP\_016880348 |
|  | XM\_005256723 |
|  | XP\_005256780 |
|  | NM\_001316332 |
|  | NP\_001303261 |
|  | NM\_145109 |
|  | NP\_659731 |
|  | XM\_017024857 |
|  | XP\_016880346 |
|  | XM\_011523959 |
|  | XP\_011522261 |
|  | NM\_002756 |
|  | NP\_002747 |
|  | XM\_017024858 |
|  | XP\_016880347 |
|  | XM\_011523958 |
|  | XP\_011522260 |
|  | NG\_028256 |
|  | AC087294 |
|  | CH879350 |
|  | EAW50386 |
|  | KF456263 |
|  | KT584604 |
|  | U66840 |
|  | AAB40653 |
|  | AB451226 |
|  | BAG70040 |
|  | AB451349 |
|  | BAG70163 |
|  | AI758302 |
|  | AK093838 |
|  | BAG52769 |
|  | AK223490 |
|  | BAD97210 |
|  | AK315505 |
|  | BAG37889 |
|  | BC032478 |
|  | AAH32478 |
|  | BM836867 |
|  | CR536514 |
|  | CAG38752 |
|  | CR541793 |
|  | CAG46592 |
|  | D87116 |
|  | BAA13248 |
|  | DA131175 |
|  | DA987047 |
|  | GQ225578 |
|  | ACT33197 |
|  | L36719 |
|  | AAC41718 |
|  | U66839 |
|  | AAB40652 |
|  | P46734 |
|  | NC\_000077 |
|  | NM\_008928 |
|  | NP\_032954 |
|  | XM\_006533368 |
|  | XP\_006533431 |
|  | NR\_157293 |
|  | AL603830 |
|  | CH466675 |
|  | EDL38689 |
|  | EDL38690 |
|  | EDL38691 |
|  | AK008141 |
|  | BAB25489 |
|  | AK011002 |
|  | BAB27321 |
|  | AK053378 |
|  | AK150067 |
|  | BAE29279 |
|  | AK159379 |
|  | BAE35034 |
|  | AK166255 |
|  | BAE38665 |
|  | AK166507 |
|  | BAE38817 |
|  | AK167209 |
|  | BAE39336 |
|  | AK167442 |
|  | BAE39528 |
|  | AK168710 |
|  | BAE40552 |
|  | AK176968 |
|  | AK179609 |
|  | AK181928 |
|  | AK185191 |
|  | AK197832 |
|  | AK219340 |
|  | BC007467 |
|  | AAH07467 |
|  | D87115 |
|  | BAA13247 |
|  | X93150 |
|  | CAA63649 |
|  | O09110 |
|  | NC\_005109 |
|  | NM\_001100674 |
|  | NP\_001094144 |
|  | AC\_000078 |
|  | AABR07029710 |
|  | AAHX01064251 |
|  | CH473948 |
|  | EDM04672 |
|  | EDM04673 |
|  | BC100095 |
|  | AAI00096 |
|  | BC160839 |
|  | AAI60839 |
|  | DY311082 |
|  | FQ228252 |
|  | FQ231052 |
|  | FQ234879 |
|  | FQ235160 |
|  | FQ235176 |
|  | XM\_005256720 |
|  | XM\_005256722 |
|  | XM\_005256721 |
|  | XR\_357655 |
|  | XR\_357654 |
|  | ABM85114 |
|  | ABBA01050464 |
|  | AC\_000149 |
|  | AAHY01093709 |
|  | CAI25794 |
|  | AAHY01093710 |
|  | AC\_000033 |
|  | AABR07029719 |
|  | XR\_357629 |
|  | AMYH02033199 |
|  | XM\_006533367 |
|  | NC\_018928 |
|  | XP\_005256779 |
|  | XP\_005256778 |
|  | XP\_005256777 |
|  | AABR06064053 |
|  | XR\_357630 |
|  | XR\_357631 |
|  | XP\_006533430 |
|  | DQ894188 |
|  | NT\_010718 |
|  | NW\_001838428 |
|  | NW\_921423 |
|  | CR597835 |
|  | Q53EZ9 |
|  | Q6FHG1 |
|  | Q6FI23 |
|  | NT\_096135 |
|  | NW\_001030465 |
|  | Q3TJG7 |
|  | Q3TLX7 |
|  | Q3TX82 |
|  | Q3UDI2 |
|  | Q5SWN9 |
|  | NW\_047334 |
|  | NW\_001084656 |
|  | Q498S1 |
|  | XM\_239239 |
|  | XP\_239239 |
|  | XM\_001077724 |
|  | XP\_001077724 |
|  | Q8BPT2 |
|  | BG077180 |
|  | AC\_000060 |
|  | NW\_926672 |
|  | AI481780 |
|  | NP\_659732 |
|  | XM\_944206 |
|  | XP\_949299 |
|  | NM\_145110 |
|  | NW\_000039 |
|  | 26343429 |
|  | 1711246 |
|  | 1711247 |
|  | 1771302 |
|  | 1771303 |
|  | 24638463 |
|  | 685174 |
|  | 1778152 |
|  | 1778153 |
|  | 24638466 |
|  | NW\_047557 |
|  | XM\_344887 |
|  | XP\_344888 |
|  | 34855852 |
|  | 34855824 |
|  | 34855825 |
|  | 34872015 |
|  | 34870815 |
|  | 34870816 |
|  | BAC35371 |
|  | 51828859 |
|  | 22094080 |
|  | 22094081 |
|  | 12842143 |
|  | 12842144 |
|  | 12846828 |
|  | 12846829 |
|  | 26343428 |
|  | 51474229 |
|  | 21618346 |
|  | 21618347 |
|  | 21618348 |
|  | 21618349 |
|  | 21618350 |
|  | 21618351 |
|  | 1778154 |
|  | 1778155 |
|  | 21752780 |
|  | 21619100 |
|  | 21619101 |
|  | 19193276 |
|  | 49168513 |
|  | 49168514 |
|  | 49456542 |
|  | 49456543 |
|  | 1711248 |
|  | 1711249 |
|  | 685173 |
|  | 13938622 |
|  | 13938623 |
|  | 123781555 |
|  | 72679727 |
|  | 123783454 |
|  | 123783894 |
|  | 123784429 |
|  | 123793297 |
|  | 56237940 |
|  | 74147536 |
|  | 74151398 |
|  | 74190987 |
|  | 74195418 |
|  | 74196955 |
|  | 74211868 |
|  | 74219944 |
|  | 81910186 |
|  | 81913477 |
|  | AW212142 |
|  | 123995025 |
|  | 62898712 |
|  | 74740344 |
|  | 74748518 |
|  | 74757561 |
|  | XM\_001130488 |
|  | XP\_001130488 |
|  | 28422418 |
|  | 47939296 |
|  | 82177618 |
|  | 82185068 |
|  | 9836504 |
|  | AAH44129 |
|  | AAH71285 |
|  | AB030899 |
|  | BAB11809 |
|  | BC044129 |
|  | BC071285 |
|  | NC\_007123 |
|  | NM\_131724 |
|  | NP\_571799 |
|  | NW\_001510789 |
|  | Q6IQW6 |
|  | Q9DGE0 |
|  | 119570771 |
|  | 148706742 |
|  | 148706743 |
|  | 148706744 |
|  | 149052855 |
|  | 149052856 |

---

|  |  |
| --- | --- |
| Swiss-Prot ID | MP2K3\_HUMAN |
|  | MP2K3\_MOUSE |
|  | Q6FI23\_HUMAN |
|  | Q5SWN9\_MOUSE |
|  | B1H230\_RAT |
|  | Q498S1\_RAT |

---

|  |  |
| --- | --- |
| Cell Localization | Cytoplasm |

---

|  |  |
| --- | --- |
| Ensembl ID | ENSG00000034152 |
|  | ENSP00000355081.5 |
|  | ENST00000361818.9 |
|  | ENSP00000345083.4 |
|  | ENST00000342679.8 |
|  | ENSP00000478619.1 |
|  | ENST00000613338.4 |
|  | ENSP00000319139.6 |
|  | ENST00000316920.10 |
|  | ENSMUSG00000018932 |
|  | ENSMUSP00000019076.3 |
|  | ENSMUST00000019076.9 |
|  | ENSRNOG00000049132 |
|  | ENSRNOP00000065373.1 |
|  | ENSRNOT00000073792.2 |
|  | ENSRNOG00000006612 |
|  | ENSP00000478619 |
|  | ENST00000361818 |
|  | ENST00000613338 |
|  | ENSMUSP00000019076 |
|  | ENSMUST00000019076 |
|  | ENSP00000345083 |
|  | ENSRNOP00000008679 |
|  | ENST00000342679 |
|  | ENSRNOT00000008679 |
|  | ENSP00000355081 |

---

|  |  |
| --- | --- |
| MGI ID | MGI:1346868 |
|  | 1346868 |
|  | 1328353 |
|  | 2144511 |

---

|  |  |
| --- | --- |
| RGD ID | 1306620 |
|  | 6492522 |
|  | 1307519 |

---

|  |  |
| --- | --- |
| Unigene ID | Mm.18494 |
|  | Hs.514012 |
|  | Rn.100064 |
|  | Hs.180533 |
|  | Rn.35084 |
|  | Dr.117346 |

---

|  |  |
| --- | --- |
| Homologene ID | 56430 |

---

|  |  |
| --- | --- |
| Shape | Sickle-vertex |

---

|  |  |
| --- | --- |
| IPI ID | IPI00220438 |
|  | IPI00218857 |
|  | IPI00218858 |
|  | IPI00114245 |
|  | IPI00230052 |
|  | IPI00911212 |
|  | IPI00886178 |

---

|  |  |
| --- | --- |
| Microarray ID | 1425456\_a\_at |
|  | A\_23\_P118422 |
|  | A\_14\_P109219 |
|  | A\_23\_P118427 |
|  | A\_24\_P296698 |
|  | 705 |
|  | OH7874 |
|  | MB174 |
|  | T68 |
|  | 1451714\_a\_at |
|  | 93313\_at |
|  | 93314\_g\_at |
|  | 93315\_at |
|  | d87115\_s\_at |
|  | A\_51\_P272822 |
|  | A\_51\_P272817 |
|  | L36719 |
|  | 215498\_s\_at |
|  | 215499\_at |
|  | 207667\_s\_at |
|  | 2075\_s\_at |
|  | D87116\_at |
|  | A\_14\_P131973 |
|  | A\_14\_P128256 |

---

|  |  |
| --- | --- |
| Homo sapiens Chromosome position | 17q11.2 |

---

|  |  |
| --- | --- |
| Ensembl Transcript ID | ENST00000342679 |
|  | ENST00000316920 |
|  | ENST00000395492 |
|  | ENST00000361818 |
|  | ENST00000395491 |

---

|  |  |
| --- | --- |
| FunctionalClass | MAP kinase kinase |
|  | protein binding |
|  | protein kinase |
|  | protein-tyrosine kinase |
|  | ATP binding |
|  | kinase |
|  | nucleotide binding |
|  | protein serine/threonine kinase |
|  | transferase |

---

|  |  |
| --- | --- |
| Hugo Symbol | MAP2K3 |

---

|  |  |
| --- | --- |
| Mus musculus Chromosome position | 11 B2 |
|  | 11 |

---

|  |  |
| --- | --- |
| KEGG pathway | Toll-like receptor signaling pathway |
|  | MAPK signaling pathway |
|  | Fc epsilon RI signaling pathway |
|  | GnRH signaling pathway |

---

|  |  |
| --- | --- |
| Rattus norvegicus Chromosome position | 10q22 |

---

|  |  |
| --- | --- |
| Ensembl Gene ID | ENSG00000034152 |

---

|  |  |
| --- | --- |
| ProteinAtlas NormalTissue | Tissue: lateral ventricle; Cell type: glial cells; Level: Negative; Expression type: Staining; Reliability: Supportive |
|  | Tissue: lung; Cell type: pneumocytes; Level: Negative; Expression type: Staining; Reliability: Supportive |
|  | Tissue: lymph node; Cell type: non-germinal center cells; Level: Weak; Expression type: Staining; Reliability: Supportive |
|  | Tissue: placenta; Cell type: decidual cells; Level: Moderate; Expression type: Staining; Reliability: Supportive |
|  | Tissue: skin; Cell type: epidermal cells; Level: Weak; Expression type: Staining; Reliability: Supportive |
|  | Tissue: kidney; Cell type: cells in tubules; Level: Moderate; Expression type: Staining; Reliability: Supportive |
|  | Tissue: ovary; Cell type: follicle cells; Level: Weak; Expression type: Staining; Reliability: Supportive |
|  | Tissue: cerebellum; Cell type: Purkinje cells; Level: Moderate; Expression type: Staining; Reliability: Supportive |
|  | Tissue: skeletal muscle; Cell type: myocytes; Level: Moderate; Expression type: Staining; Reliability: Supportive |

---
